# Loss of PopZ_*At*_ activity in *Agrobacterium tumefaciens* by Deletion or Depletion Leads to Multiple Growth Poles, Minicells, and Growth Defects

**DOI:** 10.1128/mBio.01881-17

**Published:** 2017-11-14

**Authors:** Romain Grangeon, John Zupan, Yeonji Jeon, Patricia C. Zambryski

**Affiliations:** Department of Plant and Microbial Biology, University of California Berkeley, Berkeley, California, USA; Princeton University

**Keywords:** *Agrobacterium tumefaciens*, PopZ, polar growth, riboswitch

## Abstract

*Agrobacterium tumefaciens* grows by addition of peptidoglycan (PG) at one pole of the bacterium. During the cell cycle, the cell needs to maintain two different developmental programs, one at the growth pole and another at the inert old pole. Proteins involved in this process are not yet well characterized. To further characterize the role of pole-organizing protein *A. tumefaciens* PopZ (PopZ_*At*_), we created deletions of the five PopZ_*At*_ domains and assayed their localization. In addition, we created a *popZ*_*At*_ deletion strain (Δ*popZ*_*At*_) that exhibited growth and cell division defects with ectopic growth poles and minicells, but the strain is unstable. To overcome the genetic instability, we created an inducible PopZ_*At*_ strain by replacing the native ribosome binding site with a riboswitch. Cultivated in a medium without the inducer theophylline, the cells look like Δ*popZ*_*At*_ cells, with a branching and minicell phenotype. Adding theophylline restores the wild-type (WT) cell shape. Localization experiments in the depleted strain showed that the domain enriched in proline, aspartate, and glutamate likely functions in growth pole targeting. Helical domains H3 and H4 together also mediate polar localization, but only in the presence of the WT protein, suggesting that the H3 and H4 domains multimerize with WT PopZ_*At*_, to stabilize growth pole accumulation of PopZ_*At*_.

## IMPORTANCE

*Agrobacterium tumefaciens* is a rod-shaped bacterium that grows by addition of PG at only one pole. The factors involved in maintaining cell asymmetry during the cell cycle with an inert old pole and a growing new pole are not well understood. Here we investigate the role of PopZ_*At*_, a homologue of *Caulobacter crescentus* PopZ (PopZ_*Cc*_), a protein essential in many aspects of pole identity in *C. crescentus*. We report that the loss of PopZ_*At*_ leads to the appearance of branching cells, minicells, and overall growth defects. As many plant and animal pathogens also employ polar growth, understanding this process in *A. tumefaciens* may lead to the development of new strategies to prevent the proliferation of these pathogens. In addition, studies of *A. tumefaciens* will provide new insights into the evolution of the genetic networks that regulate bacterial polar growth and cell division.

## INTRODUCTION

The alphaproteobacterium *Agrobacterium tumefaciens* is the causative agent of crown gall disease in flowering plants. During pathogenesis, *A. tumefaciens* transfers DNA via its *vir* type IV secretion system to a host plant cell, where the transferred DNA becomes stably integrated into a plant chromosome. Expression of genes on the transferred DNA ultimately leads to the production of the gall (1, 2). The ability of *A. tumefaciens* to transfer engineered DNA to a broad range of dicotyledonous plants is routinely exploited to generate transgenic plants for research or agriculture.

Recently, studies of *A. tumefaciens* have contributed to an expanded perspective on the growth of rod-shaped bacteria. *Escherichia coli* and *Bacillus subtilis*, rod-shaped bacteria that serve as model systems for growth and cell division, grow by addition of peptidoglycan (PG) in dispersed patches along the sidewalls of the entire cell length but not at the rounded ends of the cell (3). *A. tumefaciens* and other species, however, grow differently from the predominant model by addition of PG at one or, in some species, both poles of the bacteria (4–7). Two noteworthy genomic differences are correlated with these 2 modes of growth. The canonical proteins of the elongasome (which mediates dispersed growth), namely, MreB, MreC, MreD, RodA, RodZ, and PBP2, are not encoded in the *A. tumefaciens* genome (8–10), while most of the proteins of the division machinery (divisome), FtsZ, FtsA, PBP3, PBP1b, and FtsK, are conserved (8). These differences suggest that polar growth likely employs unique mechanisms to organize and regulate PG synthesis (11). It has also been suggested that some elements of the division machinery have been coopted to support polar growth (12, 13). Furthermore, many bacteria, including *Mycobacterium* and *Streptomyces* (14, 15, 16), and species in the order *Rhizobiales* (5), e.g., *Brucella* (5), *Sinorhizobium* (5), and *Agrobacterium* (4, 5, 10, 12, 17), have been identified as polar growers.

Unipolar growth in *A. tumefaciens* is part of a complex cell cycle in which (i) a new cell increases in length and diameter by addition of PG specifically at the growth pole; (ii) elongation and PG synthesis stop when the growth pole transitions to an old, nongrowing pole; (iii) a divisome is assembled which directs PG synthesis at the mid-cell; (iv) constriction of the divisome mediates septation producing 2 daughter cells; and, finally, (v) new growth poles are generated at the poles created by cell division and new polar elongation of the sibling cells begins (5, 9). *A. tumefaciens* must also replicate and segregate four genetic elements, namely, the circular chromosome, the linear chromosome, cryptic megaplasmid pAtC58, and tumor-inducing plasmid pTiC58 (2), by the time that cell division is complete (18–20). Very little is known about the spatiotemporal mechanisms that maintain the orderly progression of these events. Although it does not grow by addition of PG at the growth pole, *Caulobacter crescentus*, a member of the alphaproteobacteria, has been intensively studied for its cellular asymmetry (21). Many of the asymmetrically localized *C. crescentus* proteins are conserved in *A. tumefaciens* and may function in cellular polarization (9, 10, 17, 22).

The cellular asymmetry of *A. tumefaciens* that persists through the growth phase of the cell cycle likely requires subcellular organization and maintenance of two different programs in the cell (23), one at the growth pole and another at the inert old pole. Indeed, two homologues in *A. tumefaciens* of *C. crescentus* polar factors PopZ (PopZ_*Cc*_) (24) and PodJ_*Cc*_ (25) localize to distinct poles in *A. tumefaciens*. PopZ_*At*_ (Atu1720) localizes exclusively to the growing pole, and PodJ_*At*_ (Atu0499) localizes to the old pole and then to the new pole late in the cell cycle (9). The latter data suggest that PodJ_*At*_ may function in the transition of the growth pole to an old pole (17). In-frame deletions of *podJ_At_* (17) or *popZ_At_* (10, 22) produce major alterations in polar growth, such as branched poles and minicells.

**FIG 1:**
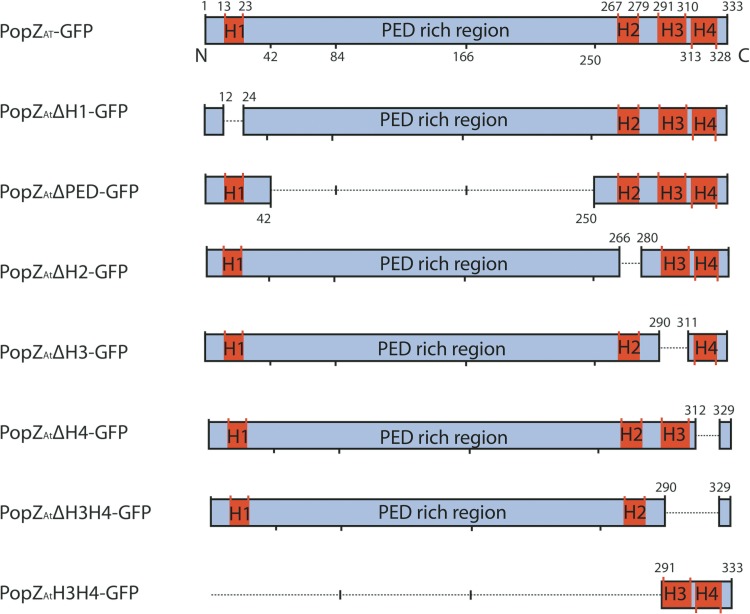
Diagram of PopZ_*At*_ deletions used in this study. The 333-amino-acid sequence of Atu1720 contains 4 α-helices (H1, H2, H3, and H4) and a disordered region rich in proline, glutamate, and aspartate (PED) as identified by Quick2D (https://toolkit.tuebingen.mpg.de/#/tools/quick2d). Deletions are shown below the full-length protein (at the top); numbers indicate the amino acids deleted in the first 6 constructs or the amino acids remaining in the bottommost construct. All regions are drawn to scale.

PopZ homologues are composed of at least 5 distinct domains: four α-helices (H1, H2, H3, and H4) and a flexible linker domain (26–28). The linker, called the PED domain and located between H1 and H2, is enriched in proline (P), glutamate (E), and aspartate (D). Although the arrangement of the four α-helical domains in the PopZ_*At*_ protein is similar to that in its *C. crescentus* homologue, the PopZ_*At*_ PED domain (~240 amino acids [aa]) is significantly longer than in PopZ_*Cc*_ (~90 aa), suggesting additional and/or different functions (Fig. 1) (9).

Here, we extend the characterization of PopZ_*At*_. First, we expressed green fluorescent protein (GFP) fusion proteins with precise deletions of the five PopZ domains and assayed their localization in wild-type (WT) cells; the results indicate that helical domains H3 and H4 are essential for polar localization. In-frame deletion of *popZ*_*At*_ caused severe growth defects with dramatically branched cells; notably, this strain was unstable. To monitor acute depletion of PopZ_*At*_, we created an inducible depletion strain by replacing the native ribosome binding site (RBS) with a riboswitch; translation occurred only with the addition of the small molecule theophylline to the medium. In the absence of theophylline, the riboswitch *popZ*_*At*_ (*RS-popZ*_*At*_) strain exhibits the same cell shape and division defects as the Δ*popZ*_*At*_ strain. We then monitored the localization of GFP-fused deletion proteins in the presence and absence of theophylline. The results show that H3 and H4 together mediate polar localization, but only in the presence of the WT protein, suggesting that the H3 and H4 domains are involved in multimerization with WT PopZ_*At*_. The PED domain likely targets the growth pole, but the H3H4 interaction is required for stable accumulation of PopZ_*At*_ at the growth pole.

## RESULTS

### Genomic context and domain structure of *popZ*_*At*_.

*popZ*_*At*_ is located on the circular chromosome of *A. tumefaciens*. The genomic context of *popZ*_*At*_ is dissimilar to the genomic context of *popZ_Cc_*, except for the presence of the *valS* gene (encoding a tRNA synthetase) a few hundred bases downstream of *popZ*_*At*_ and *popZ*_*Cc*_ (see Fig. S1 in the supplemental material).

10.1128/mBio.01881-17.1 FIG S1  *popZ* genomic context in *A. tumefaciens* and *C. crescentus*. (A) Atu numbers refer to *A. tumefaciens* strain C58 locus tags. (B) CC numbers refer to *C. crescentus* CB15N locus tags. Open reading frames (ORFs) are represented to scale. Strain *ΔpopZ*_*At*_ was created using allelic exchange to remove the PopZ_*At*_ coding sequence in frame. *fadL*, long-chain fatty acid transport protein. *valS*, valyl-tRNA synthetase. Hp, hypothetical protein. *pcm*, protein-l-isoaspartate O-methyltransferase. *pim*, protein-l-isoaspartate O-methyltransferase. *rsaFb*, type (I) *lcfaCl*, long-chain-fatty acid–CoA ligase. Download FIG S1, TIF file, 35.6 MB. Copyright © 2017 Grangeon et al. 2017 Grangeon et al. This content is distributed under the terms of the Creative Commons Attribution 4.0 International license.

PopZ_*At*_ is larger than PopZ_*Cc*_ (333 amino acids versus 177, respectively). The domain structures, however, are similar (Fig. 1). PopZ_*At*_ contains 4 predicted α-helical domains (H1, H2, H3, and H4). H1 is at the N terminus, while H2, H3, and H4 are clustered toward the C terminus (Fig. 1). Between H1 and H2, the PED domain (241 amino acids) is enriched in proline (P), glutamate (E), and aspartate (D) at 11%, 9%, and 6%, respectively (Fig. 1). In contrast, the PED domain in PopZ_*Cc*_ is only 87 amino acids in length. The specific sequence of amino acids does not appear to be important, but a minimal length and possibly an overall negative charge are required to facilitate protein-protein interactions (26). The increased length of the PED domain in PopZ_*At*_ suggests that it may participate in additional functions or interactions compared to PopZ_*Cc*_.

### Growth pole localization determinants in PopZ_*At*_ domains in WT *Agrobacterium*.

**FIG 2:**
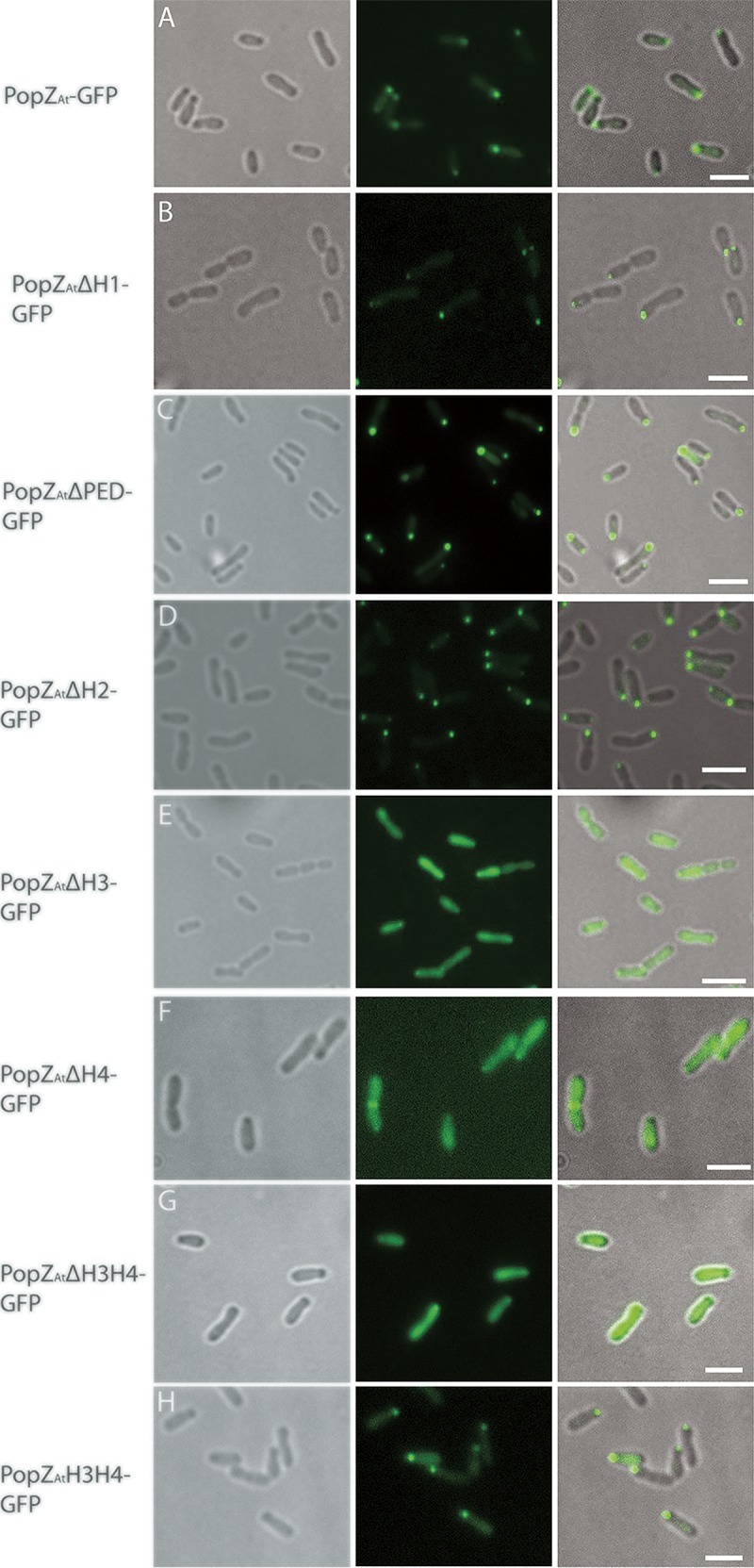
The C-terminal H3H4 domain is necessary and sufficient for polar localization of PopZ_*At*_ in WT *A. tumefaciens*. (A) Full-length PopZ_*At*_-GFP. (B) PopZ_*At*_ΔH1-GFP. (C) PopZ_*At*_ΔPED-GFP. (D) PopZ_*At*_ΔH2-GFP. (E) PopZ_*At*_ΔH3-GFP. (F) PopZ_*At*_ΔH4-GFP. (G) PopZ_*At*_ΔH3H4-GFP. (F) PopZ_*At*_H3H4-GFP. Left column, phase-contrast images; middle column, fluorescence images; right column, merged images. Scale bar = 2 μm.

To determine which domains of PopZ_*At*_ are required for polar localization and function, we cloned precise deletions of the coding sequences of these five domains into a plasmid carrying a promoter inducible with isopropyl β-d-1 thiogalactopyranoside (IPTG) (29) and in frame with the N terminus of GFP coding sequences (Fig. 1). In addition, the 43 C-terminal amino acids containing H3 and H4 were fused to GFP. All constructs were transformed into WT cells, and their expression was induced by IPTG. The subcellular localizations of the different PopZ_*At*_ deletions fused to GFP were monitored by fluorescence microscopy (Fig. 2). The images shown are representative of hundreds of cells whose localization was quantified as exhibiting polar, diffuse, or occasional bipolar localizations (Fig. S2).

10.1128/mBio.01881-17.2 FIG S2  Localization quantification for the PopZ_*At*_-GFP deletions in *A. tumefaciens* WT. Histograms represent the localization pattern for all the PopZ_*At*_-GFP deletion variants used in this study. Cells were counted and organized in 3 categories: bipolar, diffuse, and polar. Download FIG S2, TIF file, 16.5 MB. Copyright © 2017 Grangeon et al. 2017 Grangeon et al. This content is distributed under the terms of the Creative Commons Attribution 4.0 International license.

As reported previously (9, 10, 22), full-length PopZ_*At*_-GFP localized to the growth pole (Fig. 2A). PopZ_*At*_ΔH1-GFP, PopZ_*At*_ΔPED-GFP, and PopZ_*At*_ΔH2-GFP also localized to the growth pole (Fig. 2B, C, and D, respectively). In contrast, PopZ_*At*_ΔH3-GFP, PopZ_*At*_ΔH4-GFP, and PopZ_*At*_ΔH3H4-GFP exhibited diffuse fluorescence throughout the cytoplasm (Fig. 2E, F, and G, respectively). The short C-terminal fusion (PopZ_*At*_H3H4-GFP) localized to the growth pole (Fig. 2H). Thus, all deletions that retained the combination of H3 and H4 localized to the growth pole.

### Deletion of *popZ*_*At*_ causes dramatic branching and abnormal cell division.

As the GFP fusions described above were expressed in the presence of WT endogenous PopZ_*At*_, we do not know if C-terminal H3H4 can localize to the growth pole on its own or whether it localizes to the pole via protein-protein interaction. To address this issue and to characterize the role of PopZ_*At*_ in the *A. tumefaciens* cell cycle, we created a *popZ*_*At*_ knockout strain (Δ*popZ*_*At*_) where the coding sequence of PopZ_*At*_ has been deleted from the circular chromosome (Fig. S1A).

**FIG 3:**
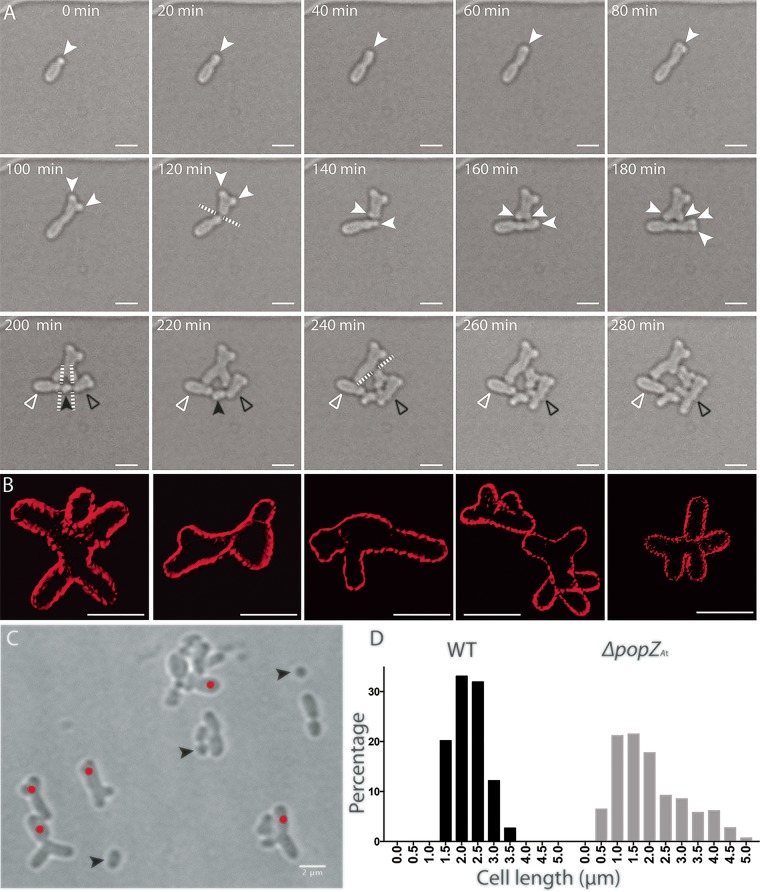
Δ*popZ*_*At*_ cells produce branched and elongated cells and minicells. (A) Time-lapse microscopy of Δ*popZ*_*At*_ cells, showing the formation of branched cells after growth pole splitting. Open white arrowheads indicate a cell that does not elongate after septation; open black arrowheads indicate a cell on the right side that is described in the text. (B) Three-dimensional reconstruction from SIM images of Δ*popZ*_*At*_ cells with membranes labeled by FM4-64. (C) A field of view of Δ*popZ*_*At*_ cells in phase-contrast microscopy showing different morphology defects. (D) Cell length distribution of WT cells (*n* = 263) and Δ*popZ*_*At*_ cells (*n* = 293). Solid white arrowheads, growth poles; solid black arrowheads, minicells; white dashed lines, plane of divisions; red dots, abnormally wide cells with polar branching. Scale bar = 2 μm.

The effect of *popZ*_*At*_ deletion on growth and cell division is best illustrated by examining images from a time-lapse series (Fig. 3A). A *ΔpopZ*_*At*_ cell transiently appears WT (Fig. 3A, 0 min). After a period of extension, the growth pole bifurcates, creating 2 growth poles (Fig. 3A, 80 min). Elongation occurs at both of these growth poles but stops when the original cell divides approximately at mid-cell (Fig. 3A, 120 to 140 min), producing a Y-shaped sibling cell (upper cell) and an unbranched sibling cell (lower cell) (Fig. 3A, 140 min). We discuss below each of the sibling cells that were present following the cell division at 140 min (Fig. 3A; see also Movie S1 in the supplemental material). Movie S2 shows a dramatic example of a cell with 6 growth poles that formed a cauliflower-like large cell.

10.1128/mBio.01881-17.6 MOVIE S1  Time-lapse microscopy of *ΔpopZ*_*At*_ cells showing the formation of branched cells after growth pole splitting. Selected frames are presented in Fig. 3A. Download MOVIE S1, AVI file, 0.3 MB. Copyright © 2017 Grangeon et al. 2017 Grangeon et al. This content is distributed under the terms of the Creative Commons Attribution 4.0 International license.

10.1128/mBio.01881-17.7 MOVIE S2  Time-lapse microscopy of *ΔpopZ*_*At*_ cells showing a dramatic example of a cell with 6 growth poles that form a cauliflower-like large cell. Download MOVIE S2, AVI file, 0.1 MB. Copyright © 2017 Grangeon et al. 2017 Grangeon et al. This content is distributed under the terms of the Creative Commons Attribution 4.0 International license.

In the upper Y-shaped cell (Fig. 3A, 140 to 220 min), the new growth pole splits to produce two growth poles, creating an X-shaped cell with two old poles and two new growth poles, which increase in length (Fig. 3A, 140 to 220 min). The growth pole extension on the right goes on to produce a small Y-shaped cell (Fig. 3A, 240 min), which then divides to produce a small Y-shaped cell whose new growth pole widens (Fig. 3A, 280 min). Thus, in the absence of *popZ*_*At*_, the growth pole splits into two active growth poles that can produce a Y-shaped cell or an X-shaped cell. Deletion of PopZ_*At*_, however, does not appear to affect the transition from growth pole to old pole or the formation of new growth poles at the division site.

The lower cell initiates two constrictions (Fig. 3A, 140 to 280 min), while the growth pole on the right side splits into two growth poles (Fig. 3A, 160 to 180 min). Elongation stops as septation occurs simultaneously at the two constriction sites (Fig. 3A, 200 min, dashed lines), producing three cells (Fig. 3A, 200 to 220 min). Remarkably, the small cell in the middle elongates (Fig. 3A, 220 to 280 min, black arrowhead). The cell on the left did not grow during the 80 min of observation (open white arrowhead); potentially, this reflects unequal partitioning of genetic elements (22). The cell produced on the right (Fig. 3A, 220 min, open black arrowhead) exhibited growth pole splitting (Fig. 3A, 240 to 280 min). Therefore, in addition to growth pole splitting, multiple sites of septation can form during a single cell cycle in the absence of PopZ*_At_*.

The gallery of superresolution structured illumination microscopy (SIM) images shown in Fig. 3B illustrates the variety of cell shapes that result from the combination of growth pole splitting and aberrant cell division. In addition, the septation that followed inaccurate placement of cell division machinery sometimes produced minicells (Fig. 3C, black arrowheads), either when cell division occurred too close to the end of the cell or when septation occurred simultaneously at two sites in close proximity. The frequent observation of long, branching cells and minicells in a population of *ΔpopZ*_*At*_ cells is reflected in a graph of their cell lengths compared to the WT cell lengths (Fig. 3D). The broader distribution of lengths in the deletion strain ranged from 0.5 μm to more than 5 μm. In contrast, WT cell lengths clustered between 1.5 μm and 3.5 μm.

### Regulation of PopZ_*At*_ activity by a riboswitch.

**FIG 4:**
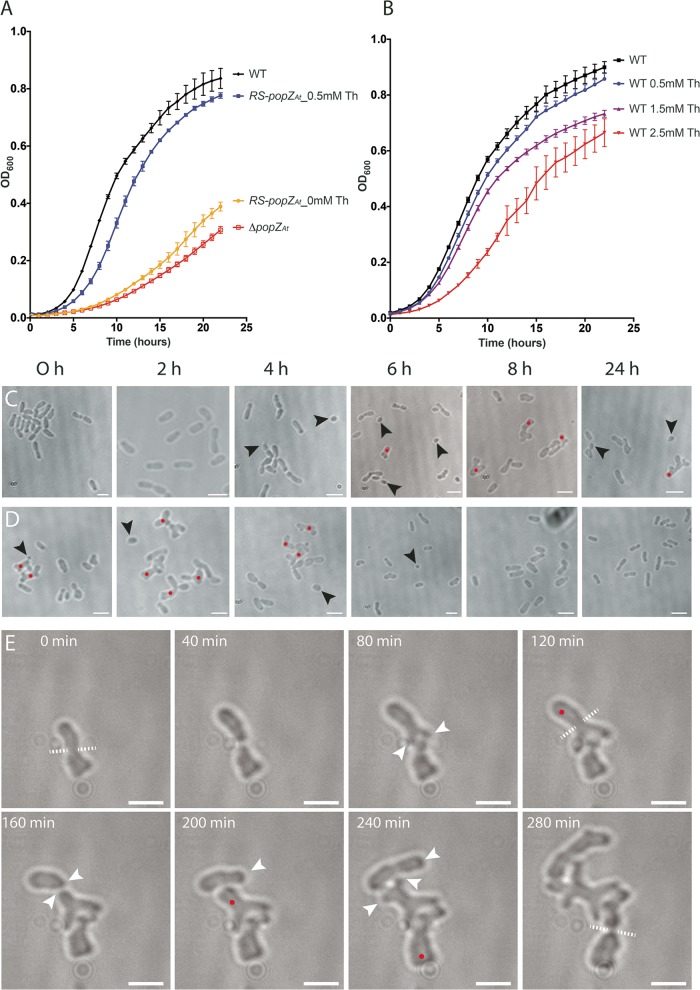
*RS-popZ*_*At*_ cells without theophylline have the same phenotype as Δ*popZ*_*At*_ cells. (A) Growth curves of WT cells (black curve), *RS-popZ*_*At*_ cells with 0.5 mM theophylline (Th; blue curve), *RS-popZ*_*At*_ cells with 0 mM theophylline (yellow curve), and ΔPopZ_*At*_ cells (red curve). (B) Growth curves of WT cells exposed to the following concentrations of theophylline: 0 mM (black curve), 0.5 mM (blue curve), 1.5 mM (purple curve), and 2.5 mM (red curve). All growth curves resulted from 4 replicates for each treatment. (C) Depletion experiment: *RS-popZ*_*At*_ strain. (D) Induction experiment: *RS-popZ*_*At*_ strain. (E) Time-lapse microscopy shows that *RS-popZ*_*At*_ cells without theophylline mimic the Δ*popZ*_*At*_ phenotype. White arrowheads, growth poles; black arrowheads, minicells; red dots, branched cells; white dashed lines, plane of division. Scale bar = 2 μm.

The growth rate of the Δ*popZ* strain was much reduced compared to that of the WT (Fig. 4A). However, repeated passage of the Δ*popZ* strain through cycles of dilution and growth in liquid culture revealed that this strain was unstable and reverted to the WT growth rate and cell morphology. This reversion was observed multiple times in independent experiments starting from single colonies, although the number of daily dilutions prior to reversion ranged between 2 and 5. As an additional copy of *popZ*_*At*_ was not found in the genome, the basis for the reversion is the subject of current investigations.

To avoid the problem of genetic instability, we created a strain (*RS*-*popZ*_*At*_) to control expression of PopZ_*At*_ that combined the native promoter and endogenous transcriptional regulation with exogenous regulation of translation. To accomplish this, the endogenous coding sequence for the RBS in *popZ*_*At*_ was replaced with a riboswitch coding sequence (30) adjacent to the start codon of *popZ_At_* (Fig. S3A and B). Transcription of this construct produced an mRNA with a secondary structure that masked the RBS and prevented translation. Binding of the small molecule theophylline to the riboswitch, however, changed the conformation of the riboswitch and allowed the ribosome to access the RBS and initiate translation (30).

10.1128/mBio.01881-17.3 FIG S3  Insertion of the riboswitch and proof of concept. (A) The sequence containing the *popZ_At_* ribosome binding site (RBS) in the circular chromosome was replaced by the riboswitch sequence. (B) The sequence containing the RBS in the plasmid pSRKGm was replaced by the riboswitch sequence. (C and D) Fluorescence images of C58 (pSRKGm:*popZ_At_-gfp*) in the absence (C) or presence (D) of IPTG. (E to G) Fluorescence images of C58 (pSRKGm:*RS-popZ_At_-gfp*) in the absence of theophylline and IPTG (E), in the absence of theophylline and presence of IPTG (F), and in the presence of both IPTG and theophylline (G). All images were taken under the same imaging conditions. In panels C, E, and F, the background fluorescence is weaker than that seen in panels D and G. In panel C, even in the absence of IPTG, we detected the weak localization of PopZ_*At*_-GFP to the pole due to the leaky expression from the *lacI* promoter. Scale bar = 2 μm. Download FIG S3, TIF file, 41.6 MB. Copyright © 2017 Grangeon et al. 2017 Grangeon et al. This content is distributed under the terms of the Creative Commons Attribution 4.0 International license.

The efficacy of this system in *A. tumefaciens* was demonstrated by first introducing the riboswitch into pSRKGm (29), which carries *popZ_At_-GFP* under the control of the *lac* operator (Fig. S3B). Without the riboswitch, expression is induced by IPTG (Fig. S3D); however, some background expression was evident without IPTG (Fig. S3C). With the additional regulation of the riboswitch, background expression and localization of PopZ_*At*_-GFP were undetectable in the absence of both IPTG and theophylline (Fig. S3E). Addition of IPTG (Fig. S3F) resulted in faint diffuse fluorescence that was barely above the background level. Addition of both IPTG and theophylline resulted in robust expression of PopZ_*At*_-GFP and specific localization to the growth pole (Fig. S3G). To optimize theophylline induction and growth, we first grew the WT strain in a range of theophylline concentrations (Fig. 4B). While the growth rate in Luria-Bertani (LB) broth supplemented with 1.5 mM and 2.5 mM theophylline was partially inhibited, growth in 0.5 mM theophylline was similar to that of the WT without theophylline (Fig. 4B). Therefore, 0.5 mM theophylline was used in all subsequent experiments. The growth rates of the Δ*popZ*_*At*_ strain and the *RS-popZ*_*At*_ strain without theophylline in liquid culture were similarly reduced (see lower curves in Fig. 4A), suggesting a lack of PopZ_*At*_ expression in the riboswitch depletion strain (minus theophylline).

After replacement of the endogenous *popZ*_*At*_ RBS with the riboswitch (Fig. S3A), we verified the utility of this strain in depletion and induction experiments. The *RS-popZ_At_* strain was grown overnight (16 h) in liquid cultures either with or without theophylline. To deplete PopZ_*At*_ and to monitor the effects of depletion on cell shape, the plus-theophylline overnight culture was diluted in LB medium without theophylline and samples were withdrawn for imaging to monitor changes in cell shape every 2 h over the following 8 h (Fig. 4C). A final sample was withdrawn after 24 h. In the depletion experiment, minicells were produced after 4 h (Fig. 4C, black arrowheads). Split growth poles began to appear after 6 h of depletion (Fig. 4C, red dots). By 8 h, many cells exhibited split growth poles. At 24 h, most cells were aberrant in shape or size. After 24 h without theophylline, the cells were very similar in appearance to *ΔpopZ*_*At*_ cells; hence, the absence of PopZ_*At*_ expression severely altered cell growth.

To induce PopZ_*At*_, the *RS-popZ*_*At*_ strain was first grown overnight without theophylline and was then diluted into LB medium with theophylline and imaged every 2 h over the following 8 h (Fig. 4D). The results show a gradual change in morphology toward a WT phenotype. After 6 h, there were a few small cells and minicells, but a clear rescue of the mutant phenotype occurred after 8 h of theophylline induction. By 24 h, the cells were completely WT in shape (Fig. 4D, 24 h).

To monitor the depletion of PopZ_*At*_ more closely, we performed time-lapse analyses. Cells were first grown for 16 h in minus-theophylline medium; we started our observations when the cell population contained predominantly split poles (Fig. 4E, labeled as 0 min in this experiment), similarly to Δ*popZ*_*At*_ cells with split growth poles (Fig. 3A, 100 min). After cell division, new growth poles are established in sibling cells at division sites; these growth poles were unstable, however, and often split into two growth poles (Fig. 4E, 80 to 240 min; see also Movie S3). As in the Δ*popZ*_*At*_ cells, misplaced constrictions (Fig. 4E, 280 min) also occurred when cells were depleted of PopZ_*At*_ in the *RS-popZ*_*At*_ strain. Results from the depletion and induction experiments as well as the time-lapse imaging indicate that the riboswitch system can confer PopZ_*At*_ activity after addition of theophylline and reduce PopZ_*At*_ activity (in the absence of theophylline) to the level seen with a deletion strain. Importantly, the *RS-popZ*_*At*_ strain is stable and allows us to better address the localization and function of the five domains of PopZ_*At*_.

10.1128/mBio.01881-17.8 MOVIE S3  Time-lapse experiment using *RS-popZ*_*A*t_ cells without theophylline, showing the formation of branched cells that mimic the Δ*popZ*_*At*_ phenotype. Selected frames are presented in Fig. 4E. Download MOVIE S3, AVI file, 0.4 MB. Copyright © 2017 Grangeon et al. 2017 Grangeon et al. This content is distributed under the terms of the Creative Commons Attribution 4.0 International license.

### Growth pole localization of PopZ_*At*_ mutants following riboswitch-controlled expression of WT PopZ*_At_*.

**FIG 5:**
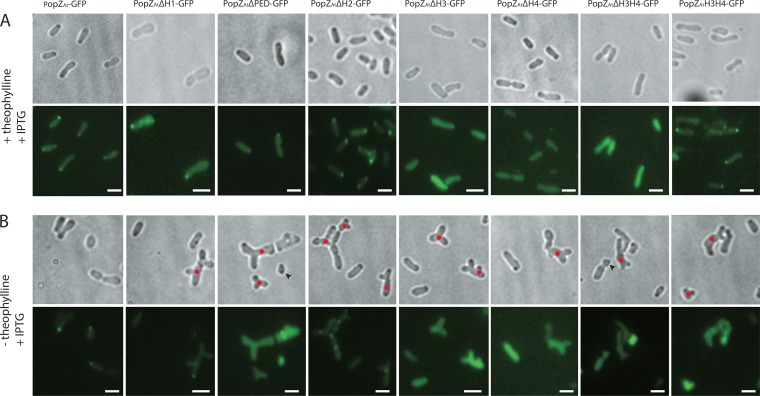
Localization of different PopZ_*At*_ deletions in *RS-popZ*_*At*_ cells grown with or without theophylline. Localization of PopZ_*At*_-GFP IPTG-inducible plasmid-borne deletion mutants expressed (A) in *trans* to WT chromosomal PopZ*_At_* in the presence of theophylline or (B) in the absence of WT chromosomal PopZ_*At*_ is indicated. Scale bar = 2 μm.

Two types of experiments were performed with the RS-*popZ_At_* strain. First, localization of PopZ_*At*_-GFP mutants was assessed by inducing their expression in the presence of full-length PopZ_*At*_ activity (induced by theophylline; Fig. 5A). Second, we determined whether growth pole localization of any PopZ_*At*_-GFP deletions occurs in the absence of full-length PopZ_*At*_ (minus theophylline; Fig. 5B).

The localization of PopZ_*At*_ mutants expressed in the presence of full-length PopZ_*At*_ (induced with theophylline) was identical to that observed under conditions of expression in WT *A. tumefaciens* (Fig. 2). PopZ_*At*_ΔH1-GFP, PopZ_*At*_ΔPED-GFP, PopZ_*At*_ΔH2-GFP, and PopZ_*At*_H3H4-GFP all localized to the growth pole (Fig. 5A). Mutants with a deletion of H3 (PopZ_*At*_ΔH3-GFP), H4 (PopZ_*At*_ΔH4-GFP), or H3H4 (PopZ_*At*_ΔH3H4-GFP) did not localize to the growth pole; however, the mutant with H3 and H4 alone in the construct deleted for the rest of PopZ_*At*_ (PopZ_*At*_H3H4-GFP) did localize to the growth pole (Fig. 5A). These data (determined with endogenous full-length PopZ_*At*_) might suggest that all domains except H1 and H2 are needed to target PopZ_*At*_ to the growth pole. However, this conclusion is not entirely supported by the results of expression in the absence of WT PopZ_*At*_, as discussed next.

PopZ_*At*_ mutants were also expressed in the absence of full-length PopZ_*At*_ (depleted by the absence of theophylline). Only PopZ_*At*_ΔH1-GFP and PopZ_*At*_ΔH2-GFP localized very weakly to the growth pole (Fig. 5B), and PopZ_*At*_ΔH2-GFP also exhibited mid-cell localizations that may correspond to new poles in recently divided cells (compare with phase-contrast images to see constriction sites). PopZ_*At*_ΔPED-GFP, PopZ_*At*_ΔH3-GFP, PopZ_*At*_ΔH4-GFP, PopZ_*At*_ΔH3H4-GFP, and PopZ_*At*_H3H4-GFP did not localize to the growth pole in the absence of full-length PopZ_*At*_. Interpretation of these data is confounded by the fact that the cells were abnormally shaped and therefore may not have exhibited pole-specific progressions through the cell cycle; indeed, none of the deletion constructs could complement the defect in cell morphology that occurred without full-length PopZ_*At*_. For growth pole localization, however, these data suggest that the PED domain plays a role in targeting to the growth pole but that its presence is not sufficient (Fig. 5). The H3H4 domain does not itself target the growth pole in the absence of full-length PopZ_*At*_ (Fig. 5B), but its growth pole localization in the presence of full-length PopZ_*At*_ (Fig. 5A) suggests that it is required for accumulation at the growth pole (see Discussion).

**FIG 6:**
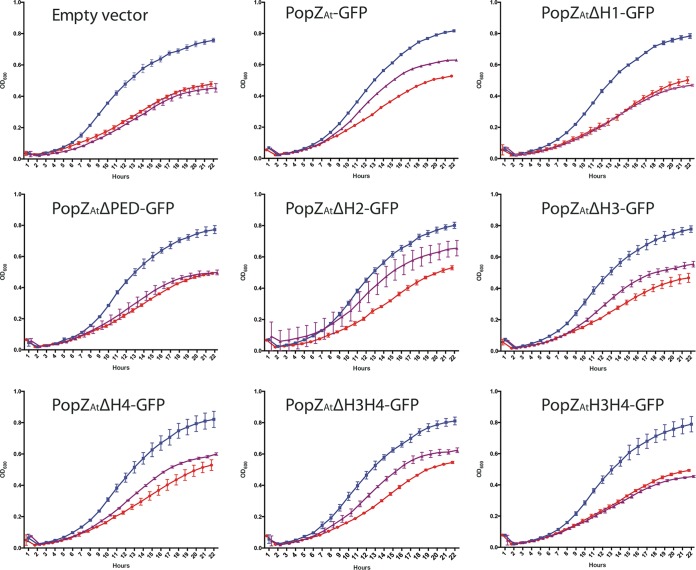
Growth assay for functional complementation of plasmid-borne PopZ_*At*_ deletion mutants. *RS-popZ*_*At*_ cells with the pSRKGm vector, empty or carrying one of the deletion mutants, were grown for 24 h and the optical density (wavelength = 600 nm) was measured every hour. Positive controls, cells grown in 0.5 mM theophylline to induce chromomosal expression of full-length PopZ_*At*_ (blue curves); negative controls, cells grown without theophylline (red curves); experimental tests of different plasmid-borne deletion constructs induced for expression by the use of 2.5 mM IPTG without theophylline (i.e., no chromosomal expression of full-length PopZ_*At*_) (purple curves). Each curve represents the results of 3 replicates.

The ability of the PopZ_*At*_ deletions to complement the loss of PopZ_*At*_ was also assayed in growth experiments (Fig. 6). Two controls were done for each of the 9 strains tested. The positive-control data (blue curves) show that all of the strains grew similarly in the presence of theophylline (i.e., full-length PopZ_*At*_ was expressed); the negative-control data (red curves) show that all of the strains grew to similarly reduced levels in the absence of theophylline (i.e., with no full-length PopZ_*At*_ expression). The experimental data (purple curves) show how well the strains can grow when the cells are expressing only (IPTG-inducible) deletions of PopZ_*At*_. Cells containing the empty vector did not grow better than the negative controls. IPTG-induced expression did not lead to full rescue by any of the constructs tested, including full-length PopZ_*At*_-GFP; this is expected since the levels of protein expressed from the plasmid-borne gene may not have corresponded to WT levels and since all proteins were fused to GFP. Both full-length PopZ*_At_*-GFP and PopZ*_At_*ΔH2-GFP partially rescue growth when PopZ*_At_* is depleted, suggesting that the H2 domain is not essential for overall cell growth. PopZ_*At*_ΔH3-GFP, PopZ_*At*_ΔH4-GFP, and PopZ_*At*_ΔH3H4-GFP gave relatively similar levels of rescue that were not quite as high as those seen with expression of full-length PopZ_*At*_-GFP or PopZ_*At*_ΔH2-GFP. The experiments performed with PopZ_*At*_ΔH1-GFP, PopZ_*At*_ΔPED-GFP, and PopZ_*At*_H3H4-GFP did not result in rescue. Note that while PopZ_*At*_ΔH3H4-GFP partially rescued growth, PopZ_*At*_H3H4-GFP (lacking H1 and the PED domains) did not rescue growth. These data together suggest that H1 (only 13 amino acids) and PED domains must both be present to restore growth.

## DISCUSSION

The cell cycle of *A. tumefaciens* comprises a number of processes, including polar growth, replication/segregation of genetic elements, cell division, and the organization of new growth poles in sibling cells. The underlying regulatory mechanisms that coordinate the *Agrobacterium* cell cycle are not understood. Nevertheless, recent work has identified factors (PopZ_*At*_ and PodJ_*At*_) that identify the growth pole versus the nongrowing old pole, respectively (9, 17). The dynamic localization of PopZ_*At*_ to the growth pole during the cell cycle suggests that this protein contributes to a growth pole-specific developmental program (9, 22). Additional data show that loss of PopZ_*At*_ impacts the segregation of the *Agrobacterium* circular chromosome and results in ectopic growth poles and multiple misplaced division sites (10, 22). Here we present detailed analyses of the functional domains of PopZ_*At*_ and a strategy for the depletion or induction of PopZ_*At*_ expression by replacing the endogenous RBS with a riboswitch.

Our results demonstrate the utility of the riboswitch for regulating the expression of endogenous genes in *A. tumefaciens*. Our system uses endogenous transcriptional mechanisms to generate *popZ*_*At*_-specific mRNA where the RBS has been replaced by a riboswitch. This mRNA cannot be translated because the riboswitch folds into a secondary structure that masks the RBS (30). The small molecule theophylline binds the riboswitch and changes the secondary structure of the mRNA to expose the RBS, allowing translation. Depletion or induction of a protein of interest is regulated by removal or addition of theophylline, respectively (30). In the present study, PopZ_*At*_ depletion in *RS-popZ*_*At*_ resulted in a phenotype identical to that of the Δ*popZ*_*At*_ mutant. Addition of theophylline restored the WT growth rate and cell shape to *RS-popZ*_*At*_ cells.

We developed the riboswitch strategy as our genetic deletion of *popZ*_*At*_ was unstable and resulted in cells growing at WT rates after only a few passages in liquid culture; such segregants preclude analyses of the exact effects of loss of *popZ*_*At*_. Thus, the riboswitch strategy is superior to that of stable genetic knockout by deletion because it allows (i) depletion of a gene that may be essential for growth and (ii) induction of protein expression while retaining the native promoter and endogenous regulation of transcription.

Localization of PopZ_*At*_ domain deletions fused to GFP expressed either alone or in *trans* to full-length PopZ_*At*_ via the riboswitch system suggests that growth pole localization of PopZ_*At*_ relies on both growth pole targeting and multimerization with WT PopZ_*At*_. Weak localization of PopZ_*At*_ΔH1-GFP and PopZ_*At*_ΔH2-GFP to the growth pole in cells without full-length PopZ_*At*_ suggests that both the PED domain and H3H4 are necessary. The PED domain is not sufficient for stable accumulation at the growth pole, however, as PopZ_*At*_ΔH3-GFP, PopZ_*At*_ΔH4-GFP, and PopZ_*At*_ΔH3H4-GFP fail to localize to the growth pole in the presence or absence of full-length PopZ_*At*_. PopZ_*At*_H3H4-GFP localizes to the growth pole only in the presence of full-length PopZ_*At*_, which suggests that H3H4 mediates growth pole localization through protein-protein interaction. Table S2 in the supplemental material summarizes these data. The most straightforward explanation is that the PED domain contains growth pole-targeting information and H3H4 mediates homo-oligomerization (26). We suggest that H3H4 stabilizes PED domain-directed localization of PopZ_*At*_ at the growth pole.

The role of PopZ_*At*_ multimerization in growth pole accumulation is suggested by the similarity between PopZ_*At*_ and PopZ_*Cc*_ in their domain structures and by the fact that both proteins are polarly localized (9, 26, 28). PopZ_*Cc*_ oscillates between the poles of *C. crescentus* and is required for pole-specific localization of at least 11 different proteins involved in chromosome segregation and cell cycle regulation (26). PopZ_*Cc*_ homo-oligomerizes, forming a matrix that is suggested to enhance polar localization of interacting proteins by limiting their diffusion (26, 28). The PopZ_*Cc*_ N-terminal 133 amino acids include two α-helices (H1 and H2) and an intervening spacer (PED domain); the latter proline rich negatively charged domain likely mediates interaction with binding partners independently of homo-oligomerization (26). H3 and H4 in the PopZ_*Cc*_ C-terminal 42 amino acids mediate homo-oligomerization (26). Overall, PopZ_*At*_ is 23% identical to PopZ_*Cc*_ in the regions that align across the entire protein (9). However, in the C-terminal 42 amino acids that contain H3 and H4, PopZ_*At*_ is 45% identical to PopZ_*Cc*_ (83% identical and similar) (9). The degree of similarity between the C-terminal domains suggests that the function of homo-oligomerization is likely conserved in PopZ_*At*_. Our data showing growth pole localization of PopZ_*At*_-H3H4 only in the presence of WT PopZ_*At*_ is consistent with this hypothesis.

The formation of numerous ectopic growth poles occurs in the absence of PopZ_*At*_. Remarkably, new poles are produced over and over again, resulting in giant groups of cells. The formation of pairs of growth poles occurs by a process consisting of widening and then splitting into two (see time-lapse images and movies); dramatic phenotypes sometimes arise where each end of a cell widens and produces two poles, resulting in four poles forming an X-shaped cell. These data suggest that PopZ_*At*_ may be a regulator of the timing and production of growth poles. The actual growth may be mediated by other cellular factors. PopZ*_At_* may regulate growth pole timing and formation by forming a mesh-like structure to sequester polar factors, as occurs with PopZ_*Cc*_ (26, 28).

Growth pole splitting was also observed in *A. tumefaciens* with a deletion of the coding sequence for PodJ_*At*_, a protein that localizes to the old pole early in the cell cycle but accumulates at the growth pole late in the cell cycle. PodJ_*At*_ has been proposed to act as a regulator of the transition of a growth pole to an old pole (17). In the Δ*podJ*_*At*_ cells, a single growth pole focus of PopZ_*At*_-GFP broadened and then divided into two foci as the growth pole split into two growth poles (see Fig. 5 in reference 17). PopZ_*At*_ is always localized in the growth pole, while PodJ_*At*_ accumulates at the growth pole in the second half of the cell cycle; thus, both proteins play direct roles in maintaining a single growth pole over the course of the cell cycle. Loss of PopZ_*At*_ activity results in substantially more growth pole splitting (producing cauliflower-like giant “cells” with numerous poles; see Movie S2 in the supplemental material) than loss of PodJ_*At*_, suggesting that PopZ_*At*_ may play a more significant role in growth pole stability.

In addition to growth pole splitting, mutants Δ*popZ*_*At*_ and Δ*podJ*_*At*_ exhibit significant problems with constriction and cell division. In both strains, cells often form multiple constrictions that do not complete septation, which may reflect problems with divisome assembly and function. That PopZ_*At*_ anchors the chromosome at the new growth pole (22) suggests that the placement, assembly, and function of the divisome at the mid-cell must be coordinated with DNA segregation. Indeed, a lack of either PopZ_*At*_ (10, 22) or PodJ_*At*_ (17) results in mislocalization of FtsZ and FtsA. Furthermore, in the Δ*podJ*_*At*_ mutant, misplaced septation often produces a minicell that does not contain DNA (17), and here we show that minicells are also produced in the Δ*popZ*_*At*_ mutant. If divisome assembly is directed to the mid-cell by a mechanism that utilizes polar markers PopZ_*At*_ and PodJ_*At*_ to detect the orientation of cellular polarity, then the absence of either protein would render this system unable to place the divisome at the mid-cell.

It is likely that the *A. tumefaciens* cell cycle employs mechanisms that integrate spatiotemporal information from both poles either to assign subcellular localization of many developmental proteins or to determine the cell cycle stage. PopZ_*At*_ likely plays multiple roles in this integration. First, it positions ParB at the growth pole; this mediates segregation of the circular chromosome (22). Second, PopZ_*At*_ is involved in positioning FtsA and FtsZ at the mid-cell, possibly a secondary effect downstream of chromosomal segregation (10, 22). Finally, the time-lapse images presented here show that, following cell division, polar growth is dependent on PopZ_*At*_ to prevent growth pole spitting. PopZ_*Cc*_ forms a mesh-like polar structure; PopZ_*At*_ may also form such a mesh that functions to stabilize the growth pole and prevent growth pole splitting.

While we have highlighted the significant similarity between PopZ_*Cc*_ and PopZ_*At*_ in their overall protein domain structures, these two proteins likely have distinct functions in their respective host cells; PopZ_*Cc*_ localizes to both poles and uses a different means of growth along all its lateral sides, whereas PopZ_*At*_ localizes only to the growth pole using an understudied means of bacterial growth from a single pole. Future work will aim to identify growth pole-localized factors that depend on PopZ_*At*_ for their sequestration and function. The riboswitch depletion strategy will be especially valuable in efforts to define essential polar growth-specific proteins.

## MATERIALS AND METHODS

### Strains and cell growth conditions.

Strains used in this study are listed in Table S1 in the supplemental material. The standard laboratory strain *A. tumefaciens* C58 containing pTiC58 (31) is our WT strain and was transformed with the relevant plasmids (Table S1) and grown in LB medium at 28°C. For time-lapse experiments, overnight cultures were diluted to 10^8^ cells/ml and grown for 4 to 5 h before imaging. Lactose-inducible expression was achieved by adding IPTG (isopropyl-β-d-thiogalactopyranoside) to cultures at a final concentration of 2.5 mM. PopZ_*At*_ expression in the *RS-popZ*_*At*_ strain was induced by addition of theophylline to overnight cultures at a final concentration of 0.5 mM, and the cultures were maintained at this concentration for the remainder of the experiment.

10.1128/mBio.01881-17.4 TABLE S1  Strains and plasmids used in this study. Download TABLE S1, DOCX file, 0.02 MB. Copyright © 2017 Grangeon et al. 2017 Grangeon et al. This content is distributed under the terms of the Creative Commons Attribution 4.0 International license.

10.1128/mBio.01881-17.5 TABLE S2  Dependence of PopZ_*At*_ growth pole localization on PED domain and H3H4 (summary of results presented in Fig. 5). Download TABLE S2, DOCX file, 0.05 MB. Copyright © 2017 Grangeon et al. 2017 Grangeon et al. This content is distributed under the terms of the Creative Commons Attribution 4.0 International license.

### Growth curves.

Growth curves were determined on a SpectraMax i3x (Molecular Devices, LLC) plate reader according to the manufacturer’s instructions. Briefly, cells were grown overnight at 28°C in LB either with or without theophylline to ensure that PopZ_*At*_ was induced or depleted, respectively, diluted to an optical density at 600 nm (OD_600_) of 0.1 in the LB with the combination of theophylline and IPTG that was used for growth curve measurement, grown for 6 h at 28°C, and diluted to an OD_600_ of 0.02 prior to transfer of 200 μl per well in a 96-well plate. Cells were grown for 24 h at 28°C with agitation, and OD_600_ was determined hourly. Each curve is the result of 3 to 4 replicates.

### Molecular cloning and strain construction.

Standard molecular cloning techniques were used to construct strains (32). All deletion mutants were generated by inverse PCR with phosphorylated primers. The *ΔpopZ*_*At*_ strain was constructed by transforming C58 with pRG023, selecting for a single crossover into the genome by growth on carbenicillin, and then selecting for a second recombination by growth on sucrose (17). The *RS-popZ*_*At*_ strain was constructed by transforming C58 with pRG040, selecting for a single crossover into the genome by growth on carbenicillin, and then selecting for a second recombination by growth on sucrose plates also containing 0.5 mM theophylline. pRG023 and pRG040 were derived from a vector created by cloning the *Bacillus subtilis sacB* gene conferring sucrose sensitivity (33) into a SacI site in the Stratagene pBluescript II SK vector, which cannot replicate in *A. tumefaciens*. The Δ*popZ*_*At*_ and *RS-popZ*_*At*_ strains were verified by PCR amplification of the relevant genomic region and sequencing.

### Time-lapse microscopy.

B04A microfluidic plates were used with a CellASIC ONIX microfluidic system (EMD Millipore) as previously described (17). Plates were flushed with LB with appropriate antibiotics and inducer for 30 min at 4 lb/in^2^. Cells (200 μl at 3 × 10^9^ cells/ml) were loaded into the microfluidics chamber from a suspension at 3 × 10^9^ cells/ml and perfused with LB with appropriate antibiotics and inducer. Cells were placed in a chamber with a ceiling height of 0.7 μm for 4 to 6 h. Cells were imaged every 10 min on an Applied Precision DeltaVision deconvolution fluorescence microscope. Images were processed using Fiji software (34).

### Fluorescence microscopy.

Cells were grown in LB or in LB with theophylline overnight, diluted in LB with IPTG or LB with theophylline and IPTG, and grown for 4 h at 28°C. Slides with agarose pads (1% agarose–phosphate-buffered saline [PBS], pH 7) were prepared. Cells were resuspended in FM4-64 for 5 min to stain cell membranes, applied to agarose pads, covered with a coverslip, and imaged on a DeltaVision microscope as described for time-lapse microscopy.

### Superresolution microscopy.

Superresolution images were captured using an Elyra PS.1 structured illumination microscope (SIM) (Carl Zeiss, Inc.) equipped with a Zeiss Plan-Apochromat 100×/1.46 oil immersion objective lens and a pco.edge scientific complementary metal-oxide semiconductor (sCMOS) camera with a 1.6× tube lens. FM4-64 fluorescence was examined with 561-nm laser excitation. The pixel size was 41 nm by 41 nm in the recorded images. Z-stacks were acquired by capturing 20 slices with a 0.1-μm step size. Three-dimensional SIM (3D-SIM) images were reconstructed using ZEN 2012 Black Edition (Carl Zeiss, Inc.) and processed with Imaris 8.1 (Bitplane Scientific).
