# Supplementary figures and images for: Loss of PopZAt activity in Agrobacterium tumefaciens by Deletion or Depletion Leads to Multiple Growth Poles, Minicells, and Growth Defects
Source: mBio. 2017 Nov 14;8(6):e01881-17. doi: 10.1128/mBio.01881-17 (PMC5686542; doi:10.1128/mBio.01881-17)

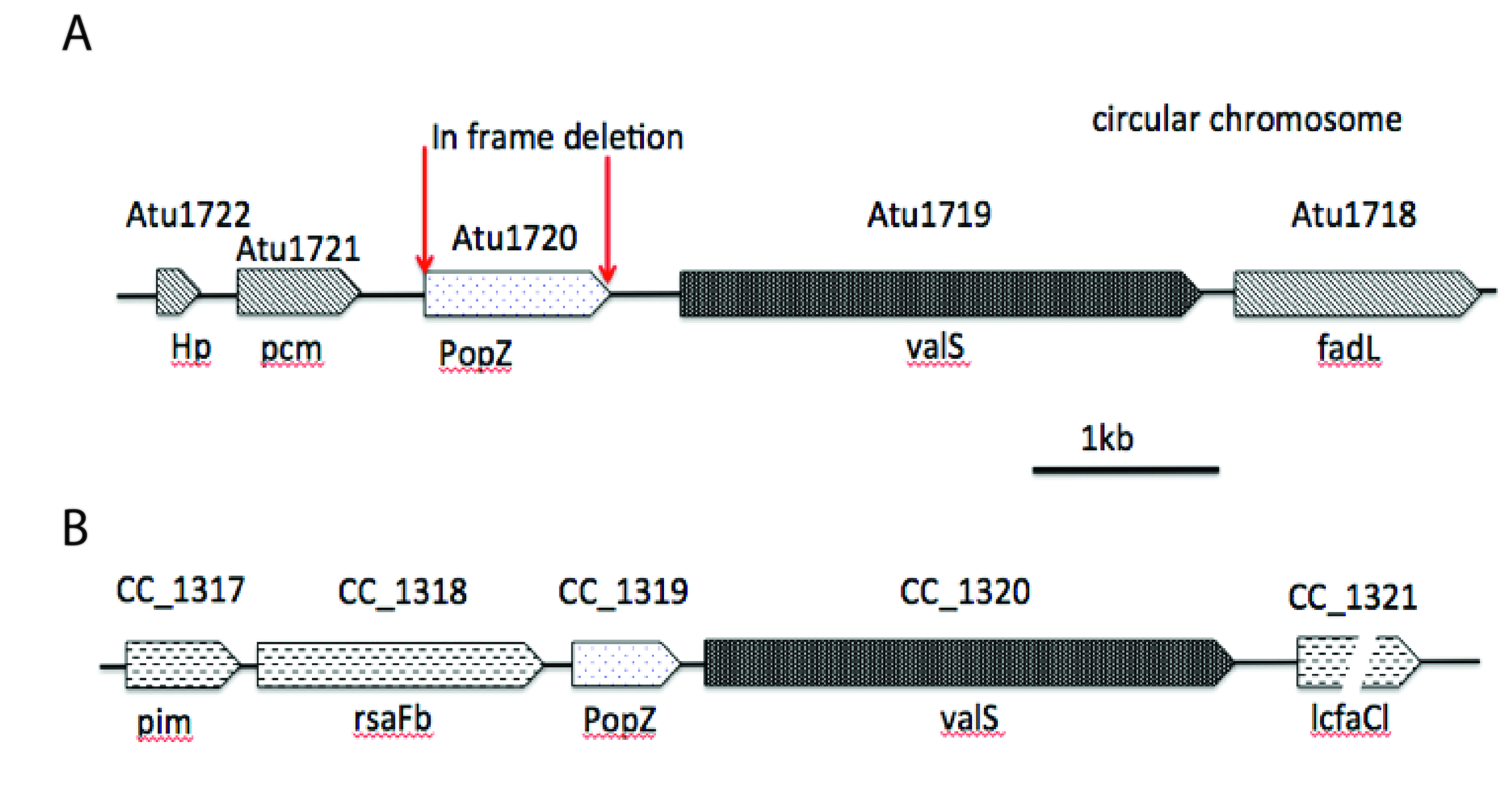

Supplement: FIG S1 [file mbo006173589sf1.tif]

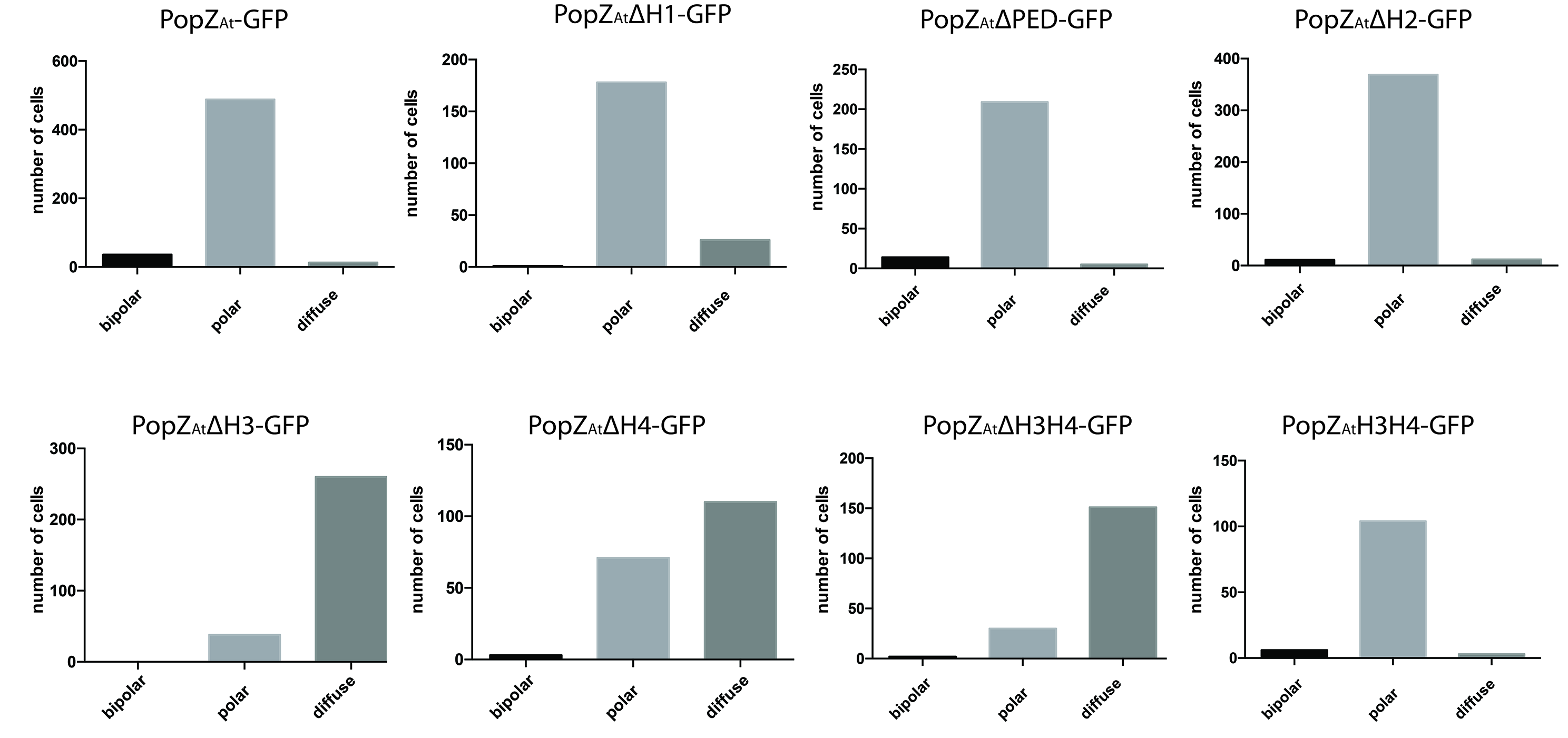

Supplement: FIG S2 [file mbo006173589sf2.tif]

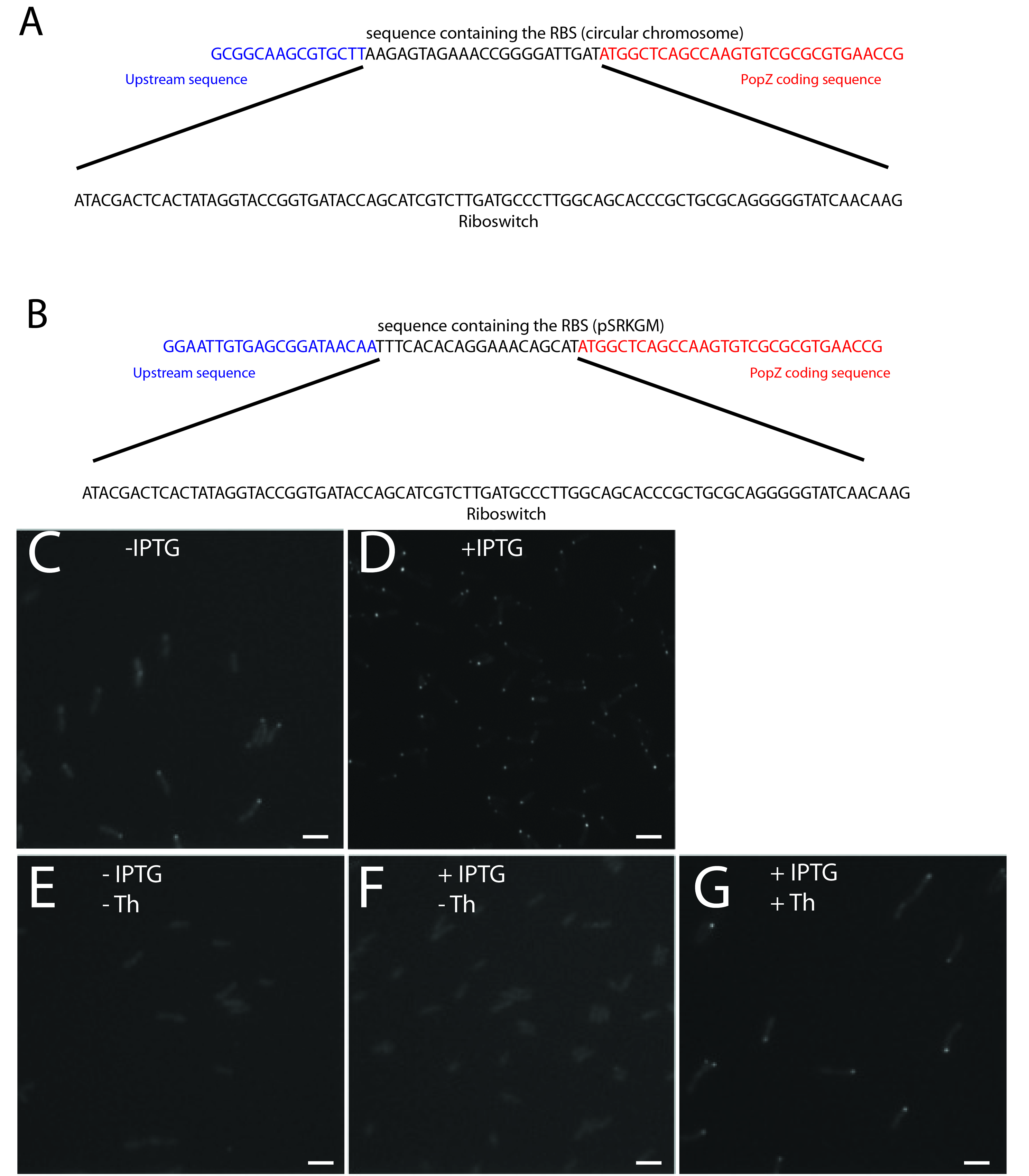

Supplement: FIG S3 [file mbo006173589sf3.tif]
