# Supplementary material for: Loss of PopZAt activity in Agrobacterium tumefaciens by Deletion or Depletion Leads to Multiple Growth Poles, Minicells, and Growth Defects
Source: mBio. 2017 Nov 14;8(6):e01881-17. doi: 10.1128/mBio.01881-17 (PMC5686542; doi:10.1128/mBio.01881-17)
Supplement: TABLE S1 [file mbo006173589st1.docx]

**Table S1.**

| \| **Strains** \| **Relevant genotype** \| **Source** \| \| --- \| --- \| --- \| \| XL1 Blue E. coli \| cloning strain, endA1 gyrA96(nal^R^ ) thi-1 recA1 relA1 lac glnV44 F'[ ::Tn10 proAB+ lacIq Δ(lacZ)M15] hsdR17(rKmK+), Tet^R^ \| Lab stock \| \| C58 \| wild-type A. tumefaciens strain C58 \| Lab stock \| \| ARG054 \| C58 Δ*popZ*, using pRG023 \| This work \| \| ARG074 \| C58 RS-*popZ*, using pRG040 \| This work \| \| **Plasmids and strains** \| **Relevant genes and construction information** \| **Source** \| \| pSRKGm \| Broad host-range, *lacI*, Gent^R^ \| (1) \| \| pBluescript II SK \| Phagemid, carbenicillin^R^ \| Stratagene \| \| pJZ156 \| pBSKII+ with sacB, carbenicillin^R^ \| (2) \| \| pRG001 \| pSRKGm with *popZ_At_-GFP*, *lacI*, gentamycin^R^ \| (3) \| \| pRG023 \| pJZ156 with 1kb sequences homologous to C58 genomic DNA flanking Atu1720 (*popZ_At_*) \| This work \| \| pRG040 \| pJZ156 with 2kb sequence homologous to C58 genomic DNA upstream *popZ_At_* and *popZ_At_* coding sequence. The RBS has been replaced by a riboswitch \| This work \| \| pRG045 \| pSRK-Gm with *popZ_At_ΔH1-gfp* \| This work \| \| pRG048 \| pSRK-Gm with *popZ_At_ΔPED-gfp* \| This work \| \| pRG051 \| pSRK-Gm with *popZ_At_ΔH2-gfp* \| This work \| \| pRG053 \| pSRK-Gm *with popZ_At_ΔH3-gfp* \| This work \| \| pRG055 \| pSRK-Gm with *popZ_At_ΔH3H4-gfp* \| This work \| \| pRG057 \| pSRK-Gm with *popZ_At_ΔH4-gfp* \| This work \| \| pRG059 \| pSRK-Gm with *popZ_At_H3H4-gfp* \| This work \| |
| --- | --- | --- | --- | --- | --- | --- | --- | --- | --- | --- | --- | --- | --- | --- | --- | --- | --- | --- | --- | --- | --- | --- | --- | --- | --- | --- | --- | --- | --- | --- | --- | --- | --- | --- | --- | --- | --- | --- | --- | --- | --- | --- | --- | --- | --- | --- | --- | --- | --- | --- | --- | --- | --- | --- | --- | --- | --- |

**SUPPLEMENTAL REFERENCES**

1. Khan SR, Gaines J, Roop RM, & Farrand SK (2008) Broad-host-range expression vectors with tightly regulated promoters and their use to examine the influence of TraR and TraM expression on Ti plasmid quorum sensing. *Appl Environ Microbiol* 74(16):5053-5062.

2. Anderson-Furgeson JC, Zupan JR, Grangeon R, & Zambryski PC (2016) Loss of PodJ in Agrobacterium tumefaciens Leads to Ectopic Polar Growth, Branching, and Reduced Cell Division. *J Bacteriol* 198(13):1883-1891.

3. Grangeon R, Zupan JR, Anderson-Furgeson J, & Zambryski PC (2015) PopZ identifies the new pole, and PodJ identifies the old pole during polar growth in Agrobacterium tumefaciens. *Proceedings of the National Academy of Sciences* 112(37):11666-11671.
