# Supplementary material for: Loss of PopZAt activity in Agrobacterium tumefaciens by Deletion or Depletion Leads to Multiple Growth Poles, Minicells, and Growth Defects
Source: mBio. 2017 Nov 14;8(6):e01881-17. doi: 10.1128/mBio.01881-17 (PMC5686542; doi:10.1128/mBio.01881-17)
Supplement: TABLE S2 [file mbo006173589st2.docx]

**Table S2**.

| Mutant | Is PED domain present in deletion? | Is H3H4 present in deletion? | Subcellular  (-Full-length) | Localization  (+Full-length) |
| --- | --- | --- | --- | --- |
| PopZ*_At_*ΔH1 | + | + | Pole | Pole |
| PopZ*_At_*ΔPED | _ | + | Cytoplasmic | Pole |
| PopZ*_At_*ΔH2 | + | + | Pole | Pole |
| PopZ*_At_*ΔH3 | + | _ | Cytoplasmic | Cytoplasmic |
| PopZ*_At_*ΔH4 | + | _ | Cytoplasmic | Cytoplasmic |
| PopZ*_At_*ΔH3H4 | + | _ | Cytoplasmic | Cytoplasmic |
| PopZ*_At_*H3H4 | - | + | Cytoplasmic | Pole |
